# Editorial: Brain Evolution: Clues From Aquatic Organisms

**DOI:** 10.3389/fnana.2021.683489

**Published:** 2021-05-07

**Authors:** Paolo de Girolamo, Jean-Pierre Bellier, Livia D'Angelo

**Affiliations:** ^1^Department of Veterinary Medicine and Animal Production, University of Naples Federico II, Naples, Italy; ^2^Molecular Neuroscience Research Center, Shiga University of Medical Science, Otsu, Japan

**Keywords:** cephalopods, agnathans, teleosts, brain structure and function, evolutionary adaptation

Aquatic species have served as models in brain evolution to address questions on the origin of the nervous system for a long time. The aquatic environment and its inhabitants have offered the possibility to understand how animals progressed from a simple nerve net to a complex centralized nervous system (Arendt et al., 2016), and how the environment has contributed to boosting the functional diversity of brain structures, shapes, and sizes, all reflecting peculiarities in species-specific sensory perception, central processing, and behavioral responses. Although fish are unequivocally the first group of organisms which come to mind when thinking about the aquatic environment, much of what we know today about brain evolution has also arisen from aquatic invertebrates, i.e., cephalopods. This collection of articles discusses topics ranging from neuropeptidergic systems responsible for different behaviors, to electric synapses formation, and neuroplasticity, in both invertebrates and vertebrates. A hierarchical clustering approach has been used to document the brain diversity of about 30 cephalopod species, correlated to the species habitats and physiological environmental adaptations, opening new avenues in evolutionary neurobiology and ecomorphology to reveal the biological basis of sensory orientation, cognitive potential, and motor abilities (Ponte et al.). Across the vertebrate tree, agnathans (lamprey) occupy the base representing intriguing models to understand early brain evolution in vertebrates. Remarkably, lamprey display complex neuropeptidergic systems and essential components of neuronal communication in the animal brain controlling and regulating several conserved behaviors, such as appetite. As an example, galanin, a neuropeptide known to regulate many physiological processes, including feeding and nociception in mammals, displays a wider expression pattern in the larval brain which is restricted to the ventral pallium, lateral hypothalamus, and prethalamus in the adult brain of sea lamprey *Petromyzon marinus* (Sobrido-Cameán et al.). Neuropeptidergic systems controlling food intake behavior are among the most studied in teleost fish, due to the high degree of conservation in vertebrates and the relevant impact in aquaculture (Amodeo et al., 2018). This is indeed the case of Atlantic Salmon (*Salmo salar* L.), a teleost species providing the unique opportunity for studying vertebrate genome evolution after an autotetraploid whole genome duplication over a period that is long enough to reveal long-term evolutionary patterns, but short enough to give a high-resolution picture of the process (Lien et al., 2016). For instance, the study on the Atlantic Salmon reports that the melanocortin-4 receptor is present as multiple paralogs (a1, a2, b1, and b2) and all paralogs are relatively well-conserved with the human homolog, sharing at least 63% amino acid sequence identity. It is worth noting that the mRNA expression of mc4r paralogs was not changed in the hypothalamus or in other highly expressed regions between the fed and fasted state of young salmon specimens (Kalananthan et al.).

Studies of the teleost brain have contributed greatly toward our understanding of the processes of brain evolution and how environmental factors, such as sensory experience, modulate brain region sizes (Hall and Tropepe). We have already learnt much about vertebrate brain evolution from the comparative neurobiological approach relying on embryonic gene expression and mature neuroanatomy, with emphasis also on brain asymmetries and lateralization (Miletto Petrazzini et al.). The combination of neuroanatomical and behavioral analyses, imaging, and cutting-edge molecular genetic techniques represents a powerful approach to investigate gene-by-environment interaction effects, how genetically encoded asymmetry may change across the lifespan, and how anatomical asymmetries are linked to behavior. Among teleost fish, zebrafish at both the larval and adult stages, are extensively used in central nervous system research by targeting various brain disorders (Stewart et al., 2014). Zebrafish possess some common (shared), as well as some specific molecular biomarkers and features of neuroglia development and neuroplasticity. It is worth noting that zebrafish do not possess typical glial-like morphology, rather they show a morphology reminiscent of astroglia. However, experiments conducted on olfactory bulbs display that these structures have a location and function similar to the mammalian astrocyte (Scheib and Byrd-Jacobs). Identification and combination of molecular markers for a specific Ca2+ store and its neuronal-type association are accurately reported in the adult zebrafish brain, where calsequestrin, a calcium binding protein, is localized on the neuronal endoplasmic reticulum (Furlan et al.). Remarkably, the two isoforms identified (Casq1 and Casq2) are differentially localized in the zebrafish brain with virtually no overlapping. In addition, they are helpful to understand adaptive neuronal function to the aquatic habitat. Moreover, zebrafish are a well-established *in vivo* model for investigating synapses within the elaborate architecture of neurons. Pieces of evidence have been discussed on the cell biological mechanisms that develop, maintain, and regulate electrical synapses and mechanistic relationships between electrical and chemical synapse formation (Martin et al.). In conclusion, neuroscientists will find useful information regarding the brains of aquatic species, and how understanding these features contributes to our understanding of brain evolution, hopefully leading to further important discoveries in both aquatic and non-aquatic neurosciences.

## Author Contributions

PG, J-PB, and LD'A contributed to the whole conception. LD'A wrote the first draft of the manuscript. All authors contributed to manuscript revision, read, and approved the submitted version.

## Conflict of Interest

The authors declare that the research was conducted in the absence of any commercial or financial relationships that could be construed as a potential conflict of interest.
